# Horticultural Therapy Reduces Biomarkers of Immunosenescence and Inflammaging in Community-Dwelling Older Adults: A Feasibility Pilot Randomized Controlled Trial

**DOI:** 10.1093/gerona/glaa271

**Published:** 2020-10-18

**Authors:** Glenn Choon Lim Wong, Ted Kheng Siang Ng, Jia Le Lee, Pei Yi Lim, Sean Kai Jie Chua, Crystal Tan, Michelle Chua, Janice Tan, Samantha Lee, Angelia Sia, Maxel K W Ng, Rathi Mahendran, Ee Heok Kua, Roger C M Ho, Anis Larbi

**Affiliations:** 1 Singapore Immunology Network, Agency for Science, Technology and Research; 2 Department of Psychological Medicine, Yong Loo Lin School of Medicine, National University of Singapore; 3 Department of Biological Sciences, Singapore Polytechnic; 4 Centre for Urban Greenery and Ecology Research, National Parks Board, Singapore; 5 Horticulture & Community Gardening Division, National Parks Board, Singapore; 6 Department of Psychological Medicine, National University Hospital, Singapore

**Keywords:** CTLA4, Geroscience, IL-6, Immunosenescence, Inflammaging

## Abstract

**Background:**

With the challenges that aging populations pose to health care, interventions that facilitate alleviation of age-related morbidities are imperative. A prominent risk factor for developing age-related morbidities is immunosenescence, characterized by increased chronic low-grade inflammation, resulting in T-cell exhaustion and senescence. Contact with nature and associated physical activities have been shown to boost immunity in older adults and may be promoted in the form of horticultural therapy (HT). We aimed to examine the effects of HT on immunosenescence.

**Method:**

We conducted a randomized controlled trial with 59 older adults assigned to either the HT intervention or waitlist control group. Older adults in the HT intervention group underwent HT intervention program over 6 months. Venous blood was drawn at baseline and at the third and sixth month from the commencement of this study. For participants who attended all 3 blood collection time points (HT: *n* = 22; waitlist: *n* = 24), flow cytometry analysis was performed on whole blood samples to evaluate the kinetics of lymphocyte subsets over the intervention period, revealing the composition of CD4^+^ and CD8^+^ subsets expressing exhaustion markers—CD57, CTLA4, and KLRG1. Enzyme-linked immunosorbent assays were employed to measure changes in plasma IL-6 levels.

**Results:**

HT is associated with increased numbers of naive CD8^+^ T cells and fewer CTLA4-expressing terminally differentiated effector CD4^+^ and CD8^+^ memory T cells re-expressing CD45RA (TEMRA). Furthermore, IL-6 levels were reduced during HT, and the frequencies of naive and TEMRA CD8^+^ T cells were found to be associated with IL-6 levels.

**Conclusion:**

HT is associated with a reduction in the levels of biomarkers that measure the extent of T-cell exhaustion and inflammaging in older adults. The positive effects of HT on T-cell exhaustion were associated with the reduction of IL-6 levels.

Population aging and the concomitant increase in incidence of age-related morbidities have necessitated interventions that target biological mechanisms of aging to improve health span by reducing age-related morbidities (1–4). In particular, changes that occur to the immune system during aging—phenomena described by the term “immunosenescence”—has been widely studied. While these changes may have heterogenous effects among individuals, the functional decline of the immune system has generally been associated with aging (5). Hallmarks of immunosenescence include weak response to novel antigens, poor recall responses, and a chronic state of low-grade inflammation known as “inflammaging.” These in turn contribute to higher rates of infection, cancer, and inflammatory diseases (6). The identification of biomarkers that relate to these age-associated immunological perturbations has allowed the evaluation of interventions that aim to alleviate the aging immune system.

Aging of the T-cell compartment is fuelled by key systemic changes. First, thymic involution contributes to the age-dependent loss of naive T cells representation in the periphery (7). This loss of naive T-cell renewal restricts the host T-cell receptor (TCR) repertoire, and is implicated in a loss of responsiveness towards against neo-antigens and vaccines in the older adults as compared to younger adults (8,9). Next, the older immune system presents higher proportions of terminally differentiated memory T cells that accumulate from continuous lifelong antigenic exposure. While these memory T cells are critical for our response against previously encountered pathogens and/or latent infections, chronic exposure to high antigenic load can induce the functional exhaustion of T cells, which is characterized by a gradual loss of T-cell effector function and expression of inhibitory receptors such as programmed cell death protein 1 (PD-1), cytotoxic T-lymphocyte-associated protein 4 (CTLA4), T-cell immunoglobulin domain and mucin domain protein 3 (TIM3), and lymphocyte activation gene 3 (LAG3) (10). The reestablishment of cytomegalovirus (CMV) chronicity in the older adults further exacerbates T-cell exhaustion and replicative senescence (11,12).

Studies on T-cell maturation have revealed phenotypic markers (CD45RA, CD27, and CD28) that can be used to identify distinct T-cell subsets—naive, central (Tcm), and effector memory (Tem) as well as terminally differentiated TEMRA cells (13). Furthermore, markers such as CD57, KLRG1, and CTLA4 expression can be used to classify senescent and exhausted T cells (14). CD57 expression, for example, is highly associated with impaired proliferative capacity in T cells, even though its function remains unknown (14). Killer cell lectin-like receptor G1 (KLRG1), a C-type lectin inhibitory receptor, is similarly linked to proliferative defects in T cells (15). On the other hand, markers of T-cell exhaustion include PD-1 and CTLA4. Both PD-1 and CTLA4 are inhibitory receptors, and engagement of their ligands, PD-L1 and CD80/86, respectively, blocks activation signals normally initiated by TCRs and CD28 costimulation (16,17).

In addition to phenomena already described, levels of inflammatory cytokines are also elevated in the serum of the older adults. This is the result of an age-dependent adaptation that sees an increased production of inflammatory molecules, including tumor necrosis factor alpha (TNF-α) and interleukin-6 (IL-6) by innate immune cells and nonimmune cell types (8,9). Existing data suggest that T-cell immunosenescence and inflammaging are closely linked, where due to bystander activation, inflammaging fuels the rate of T-cell immunosenescence and vice versa—cascading into an inflammatory milieu that gives rise to tissue damage and increases the risk of age-related morbidities (8,9,12).

Current strategies that address immunosenescence include IL-7 therapy, the use of telomerase activators, autologous naive T-cell transfusion, and physical removal of senescent T cells from circulation (18). However, the cost and invasiveness of these protocols render them impractical for implementation at a population level. Thus, cost-effective and measures with high adherence should be encouraged to promote healthy aging across populations (3,4,19). A psychosocial intervention that has gained traction among the older adults is horticultural therapy (HT). HT is a guided nature-based experience that involves park visits and the performance of gardening activities. The diverse benefits of green spaces on mental wellness have been well established, where regular exposure to nature and the engagement of physical activity in natural environments has been found to contribute to reduced stress and risk of depression (20–23).

Although the mechanisms of these benefits have not been studied, exposure to green spaces is also associated with enhanced cognitive function, including prolonged attentiveness and better working memory performance (24,25). Other subjective effects of exposure to green spaces include improved vigor, friendliness, social cohesion, and lower total mood disturbance and ratings of perceived exertion. Contact with nature also confers physical health benefits either directly or indirectly by promoting physical activity or increasing exposure to better air quality that carry lower concentrations of pollutants (26–28). A previous study on HT on individuals has suggested that HT improves the cognitive and psychosocial well-being of participants (22). HT also involves physical activity that may benefit the immunological profiles of older adults, with growing evidence suggesting that physical activity can help to boost older adults’ immunity (29,30). Furthermore, epidemiological studies showed that built environment and green space could be related to inflammation. While most of the HT studies focused on its psychosocial benefits, whether HT modulates the distribution of immune cells in older adults remains to be investigated (31). We investigated this hypothesis in the specific context of T-cell aging using a randomized controlled trial (RCT) of a HT intervention program and evaluated the effects of HT on T-cell senescence and differentiation, as well as changes to plasma biomarkers in 59 community-dwelling older adults.

## Materials and Methods

### Ethics Statement

This study was approved by the National University of Singapore Institutional Review Board (NUS IRB-Reference Code: B-15–016) and registered with clinicaltrials.gov, with the identifier: NCT02495194 (https://clinicaltrials.gov/ct2/show/NCT02495194).

### Participants

This study aimed to recruit community-dwelling older adults aged between 60 and 85 years. Older adults included in the RCT scored a minimum of 22 points on the Montreal Cognitive Assessment (MoCA) and retained the ability to provide informed consent. Older adults with severe psychiatric disorders and those with a medical history of stroke, epilepsy, ischemic heart disease, heart failure, chronic obstructive pulmonary disease, cancer, liver failure, and thyroid disorder were excluded. To ensure high capacity to participate in the intervention, older adults with marked upper and lower limb motor difficulties, and significant visual or hearing impairment were also excluded. Lastly, those undergoing any concurrent therapy, including consumption of medication(s), were also excluded.

### Intervention

This was a 2-arm, single-blind, feasibility pilot RCT. Upon collection of baseline measurements, participants were randomized into 2 groups: the HT intervention or waitlist control group. The HT intervention consists of 15 hourly sessions over 6 months. HT sessions took place weekly in the first 3 months (Sessions 1–12) and monthly (Sessions 13–15) in the remaining 3 months (Supplementary Figure 1). The 3-month follow-up intervals were decided based on a study by Gonzalez et al., whose team found that symptoms of depression were alleviated in participants after 3 months and these improvements remained sustained in the final 6-month follow-up (22). As such, the inclusion of monthly sessions beyond the initial 3 months allowed us to determine whether HT could promote long-term changes in the immune system and also address the general paucity of evidence on the long-term effects of nature-based interventions (32). Conducted by a trained practitioner and volunteer facilitators, the intervention is designed to cultivate an interest in gardening and promote relaxation. These facilitators were staff from the National Parks Board (NPB).

All participants engaged in stretching and breathing exercises for approximately 5 minutes before the commencement of any horticultural activity to promote warming of muscles and improve flexibility for subsequent physical activities. The intervention focuses on 3 core activities: (i) indoor horticulture, which aims to inspire appreciation towards nature and encourage gardening at home; (ii) park visits, which provide knowledge of various plants and landscapes; and (iii) outdoor gardening, which includes weeding, seed sowing, and education on herbal plants and fertilizer making (Supplementary Table 1).

We divided the HT participants into groups of 7 during the first session. The participants stayed with the same group members and facilitator throughout the intervention to facilitate rapport building and cohesion. Two scales examining social connectedness, the friendship scale and the positive relations with others subscale, were administered to determine if participants were more socially connected at the end of the intervention.

While physical activity has been proposed to be an important mediator of the positive effects associated with nature-based intervention, there was no consensus on whether such findings were conclusive at the conception of this HT protocol (32–36). As such, our protocol did not include any measurement of the level of physical activity involved. Venous blood samples were collected thrice, each separated by a 3-month interval and corresponding to the 3 time points within the study (baseline, third month, and sixth month). No activities were planned for the waitlist control group during this period. For ethical reasons, HT was planned for the waitlist control group after the HT intervention group completed HT and assigned assessments. While the waitlist group was subsequently enlisted for HT, no further blood samples were collected from them (Supplementary Figure 1). More information on the methodology of this specific HT intervention has been published in a detailed protocol (37).

### Data and Sample Collection

Three weeks prior to the commencement of HT, sociodemographic data (age, gender, years of education, vital signs, and body mass index [BMI]), medical history, gardening, and park visits habits were obtained from participants through a structured questionnaire. Venepunctures were performed by qualified research nurses at the research center at the 3 time points (baseline, third month, and sixth month). Venous blood was collected in cell preparation tubes (CPTs) with sodium citrate (BD plastic vacutainer #362761) for the collection of peripheral blood mononuclear cells (PBMCs) and plasma for bio-banking. All samples were delivered and processed within 3 hours after the first venepuncture of the day.

### Sample Preparation

A 22.5 µL master mix of fluorochrome-tagged antibodies (Supplementary Table 2) was prepared and aliquoted into each BD Trucount Absolute Counting Tube (BD:340034) (BD Biosciences, San Jose, CA) containing beads with known bead count. Next, 100 µL of whole blood from each sample was transferred from CPT tubes into each pre-labeled tube and mixed gently by vortexing. The tubes were then incubated in the dark at room temperature for 15 minutes before 900 µL of 1× BD FACS Lysing Solution (BD Biosciences) was added. The tubes were again capped and vortexed gently to mix the solution before incubating in the dark for 15 minutes at room temperature to lyse all red blood cells. The stained cells were then acquired using BD LSRII Fortessa flow cytometer.

### Analyses of Immunological Profiling on T-Cell Subsets

FlowJo was used to analyze the flow cytometry data. The gating strategy for various T-cell subsets can be found in Supplementary Figure 1. Percentages were converted to cell counts based on the ratio of the beads found in Trucount tubes versus the number of beads collected during acquisition on flow cytometer. Since the number of subjects lost to follow-up were relatively small, we employed per-protocol analyses. Results of participants who have missed at least one laboratory assessment were excluded from the analysis (HT group, *n* = 22, 7 exclusions; waitlist group, *n* = 24, 6 exclusions).

In the initial t-distributed stochastic neighbor embedding (t-SNE) analysis, 10 participants were selected at random from both HT intervention and waitlist control groups and 10 000 cells CD3^+^ T cells were collected to represent each time point. These 10 samples for each time point and group were concatenated to form a representative sample of 100 000 cells before undergoing further analysis by t-SNE. t-SNE served as an initial analysis tool, where multidimensional data are projected into 2-dimensional space (t-SNE1 and t-SNE2) by performing repeated pairwise comparisons of randomly selected cells and grouping them based on marker expression, ultimately clustering closely related cells. The percentages of CD8 TEMRA cells expressing different combinations of CTLA4, KLRG1, and CD57 were further analyzed by Simplified Presentation of Incredibly Complex Evaluations (SPICE) to observe changes in the expression of exhaustion markers for each study group.

### Enzyme-Linked Immunosorbent Assay Measurements of Plasma Biomarkers

To obtain plasma, whole blood samples were centrifuged at 1650*g* for 25 minutes at room temperature. Subsequently, plasma samples from the 3 time points were bio-banked at −80 °C until study completion. Assays for each biomarker were run on the same day after study completion to avoid batch effect. We employed commercially available enzyme-linked immunosorbent assay (ELISA) kits to measure the level of 9 plasma biomarkers, namely IL-6, IL-1β (ThermoFisher, Waltham, MA), CXCL12/SDF-1α, CXCL5/RANTES (R&D Systems Inc., Minneapolis, MN), BDNF (Promega Corporation, Madison, WI), DHEA (CUSABIO, Houston, TX), cortisol, hs-CRP (Tecan, Männedorf, Switzerland), and sgp130 (RayBiotech Inc., Norcross, GA).

### Statistical Analyses

Statistical analyses were performed using Graphpad Prism version 7. A *p*-value of <.05 was considered statistically significant. Friedman test was applied to HT or waitlist control groups to analyze differences within treatment groups over the 3 time points. Pearson correlation was used to examine the relationship between IL-6 concentration and subsets of CD8^+^ and CD4^+^ T cells.

## Results

### Baseline Characteristics

**Figure 1.**
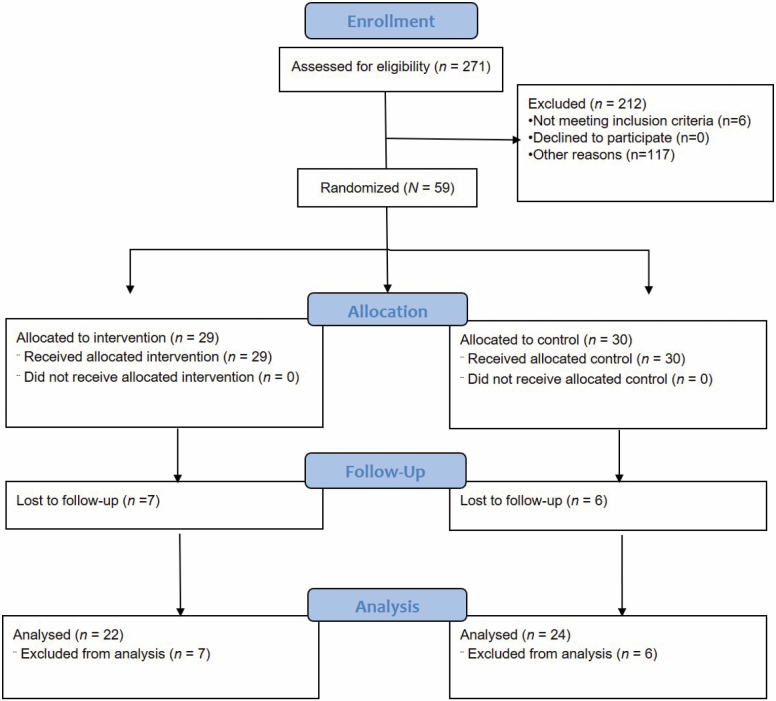
CONSORT 2010 flow diagram for horticultural therapy randomized controlled trial (HT-RCT).

We recruited 59 community-dwelling older adults, between the ages of 61 and 77 (mean = 67.1 years, *SD* = 4.31) from a neighborhood in the western region of Singapore. Of 59 participants that were included in this study, 29 were allocated to the waitlist control group and 30 to the HT intervention group (Figure 1). There were no significant differences in age, gender, ethnicity, vital signs such as blood pressures and pulse, and BMI (*p* > .05). Likewise, the frequency of morbidities and plasma biomarker levels showed no significant differences (*p* > .05) (Table 1). More than 80% of HT participants attended each intervention session.

**Table 1.** Comparison of the Baseline Characteristics Between the Participants in the Horticultural Therapy and Waitlist Control (N = 59)

Variables	Active Treatment (n = 29)		Waitlist Control (n = 30)		Test (*p*-value)
	Mean (*SD*)	n (%)	Mean (*SD*)	n (%)	
Age	67.21 (4.52)		67.00 (4.18)		*t* = 0.18 (.86)
Gender					
Male		6 (20.7%)		7 (23.30)	χ ^2^ = 0.60 (.81)
Female		23 (79.3%)		23 (76.7%)	
Years of formal education	7.34 (3.89)		7.23 (3.47)		*t* = 0.12 (.91)
BP (systolic), mm Hg	131.93 (18.52)		130.90 (14.56)		*t* = 0.24 (.81)
BP (diastolic), mm Hg	73.93 (11.35)		73.27 (9.14)		*t* = 0.25 (.81)
Pulse rate, BPM	70.10 (10.84)		67.97 (10.24)		*t* = 0.77 (.44)
BMI, kg/m^2^	24.37 (3.99)		23.34 (3.31)		*t* = 1.08 (.28)
Ethnicity					
Chinese		27 (93.1%)		30 (100%)	*F* = 2.14 (.24)
Indian		2 (6.9%)		0 (0%)	
Others		0 (0%)		0 (0%)	
Morbidities					
High blood pressure		6 (33.3%)		12 (40%)	χ ^2^ = 2.59 (.10)
Diabetes		3 (10.3%)		6 (20%)	χ ^2^ = 1.06 (.30)
Had heart attack		0 (0%)		2 (3.4%)	χ ^2^ = 2 (.16)
Arthritis		5 (17.2%)		2 (6.7%)	χ ^2^ = 1.58 (.21)
Depression		3 (10.3%)		2 (6.7%)	χ ^2^ = 0.26 (.61)
Regular park visitor					
Yes		16 (55.2%)		15 (50%)	χ ^2^ = 0.16 (.69)
No		13 (44.8%)		15 (50%)	
Perform regular gardening works					
Yes		16 (55.2%)		13 (43.3%)	χ ^2^ = 0.83 (.36)
No		13 (44.8%)		17 (56.7%)	
Baseline biomarker levels					
IL-6, pg/mL	0.98 (0.57)		1.13 (1.52)		*t* = −0.50 (.62)
IL-1β, pg/mL	0.11 (0.01)		0.12 (0.28)		*t* = −0.28 (.78)
CXCL12 (SDF-1α), pg/mL	2370.04 (500.03)		2443.70 (565.59)		*t* = −0.52 (.61)
CXCL5 (RANTES), pg/mL	2281.60 (754.21)		2403.73 (1127.37)		*t* = −0.47 (.64)
BDNF, pg/mL	576.23 (356.09)		504.56 (307.44)		*t* = 0.81 (.42)
Sgp130, pg/mL	170.13 (92.65)		158.27 (51.83)		*t* = 0.58 (.56)
Cortisol, ng/mL^a^	83.05 (30.36)		94.37 (29.21)		*t* = −1.41 (.17)
DHEA, ng/mL^a^	2.40 (0.47)		2.39 (0.43)		*t* = 0.27 (.79)
hs-CRP, µg/mL^a^	1.10 (0.48)		0.98 (0.43)		*t* = 0.62 (.54)

*Notes*: BDNF = brain-derived neurotrophic factor; BMI = body mass index; BP = blood pressure; DHEA = dehydroepiandrosterone; hs-CRP = high-sensitivity C-reactive protein; IL = interleukin.

^a^Log-transformed variables.

### t-SNE Analysis to Identify Main T-Cell Subsets Altered by HT

The 11 cellular markers used in our phenotyping panel facilitate the study of multiple subsets—we employed t-SNE as a first strategy to cluster cells based on the combinatorial expression of these markers, and to visualize how these clusters behaved in the HT intervention and waitlist groups. Due to the computationally intensive nature of this approach, only 10 participants from each group were randomly selected, and their T cells were pooled using the gating strategy shown in Supplementary Figure 1 to represent their allocated arms.

**Figure 2.**
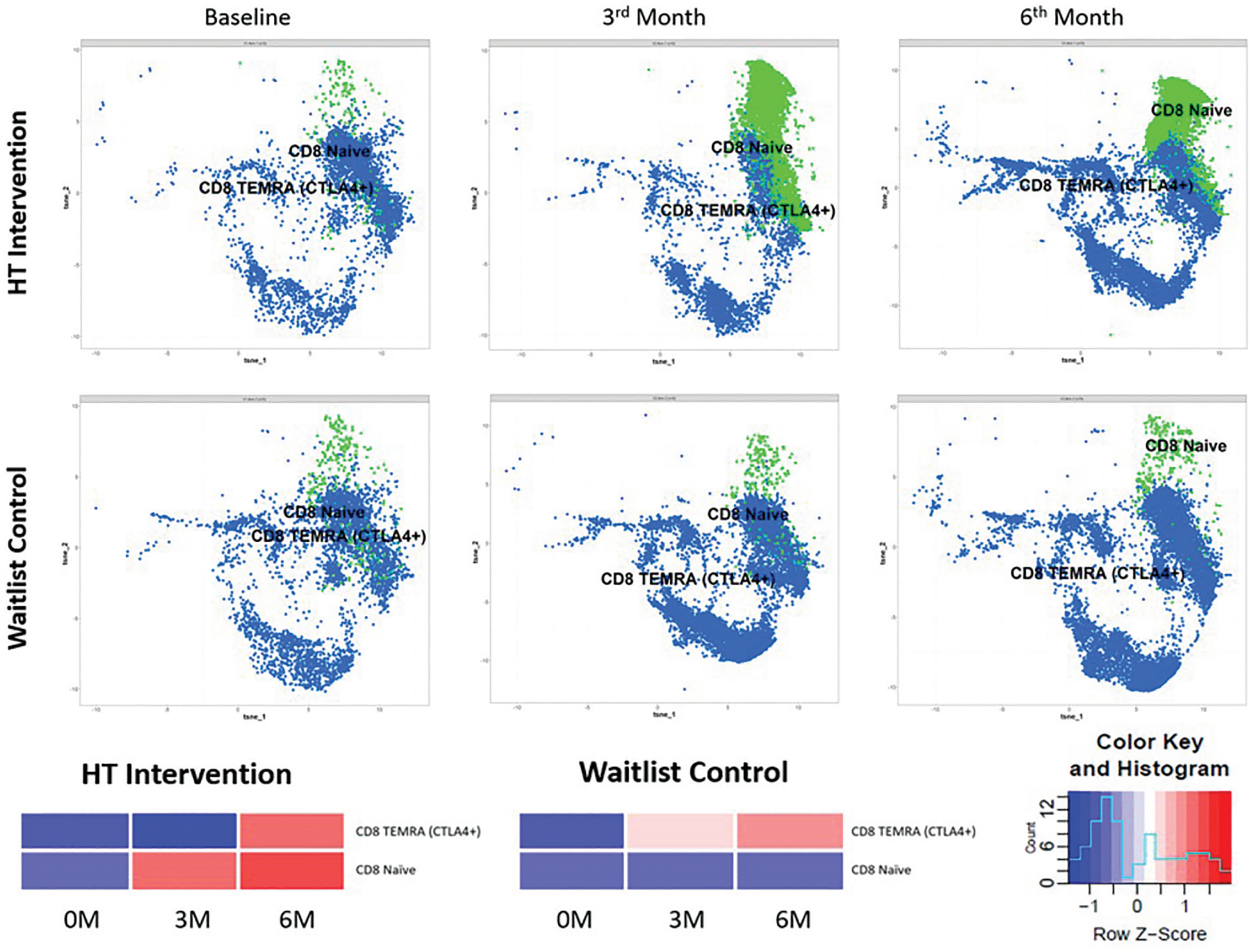
Kinetics of T-cell clusters at each time point. Graphic from t-distributed stochastic neighbor embedding (t-SNE) depicting clusters corresponding to CD8 naive and CTLA4^+^ CD8 TEMRA are shown. Naive CD8^+^ T cells (green) and CD8^+^ TEMRA cells expressing CTLA4 (blue); each dot represents a cell (bottom). The data representing these 2 T-cell clusters are presented as a heatmap (row normalized).

From pooled samples, 13 clusters were identified by t-SNE and changes in the distribution of each cluster within both HT intervention and waitlist control groups are presented in a heat map (Supplementary Figure 2). We observed consistent follow-up changes in the distribution of CD8^+^ T-cell between HT intervention and waitlist control groups, such as a marked increase in clusters which corresponded to a naive CD8^+^ T-cell (highest CD45RA, CD27, and CD28 expression) phenotype following HT as compared to the control group. We also observed that the accumulation of CD8^+^ TEMRA cells expressing CTLA4 was more prominent in the control group at the 3M time point than the HT group, although the proportion of these cells were similar in both groups at the 6M time point. A simplified figure depicting only the kinetics of these 2 clusters for each time point and the corresponding heat map is shown in Figure 2. From Supplementary Figure 2B, we did not observe kinetics in the naive and TEMRA CD4^+^ subsets that were uniquely prominent for each allocation arm.

### Absolute Numbers of Naive CD8 T Cells Increase During HT

**Figure 3.**
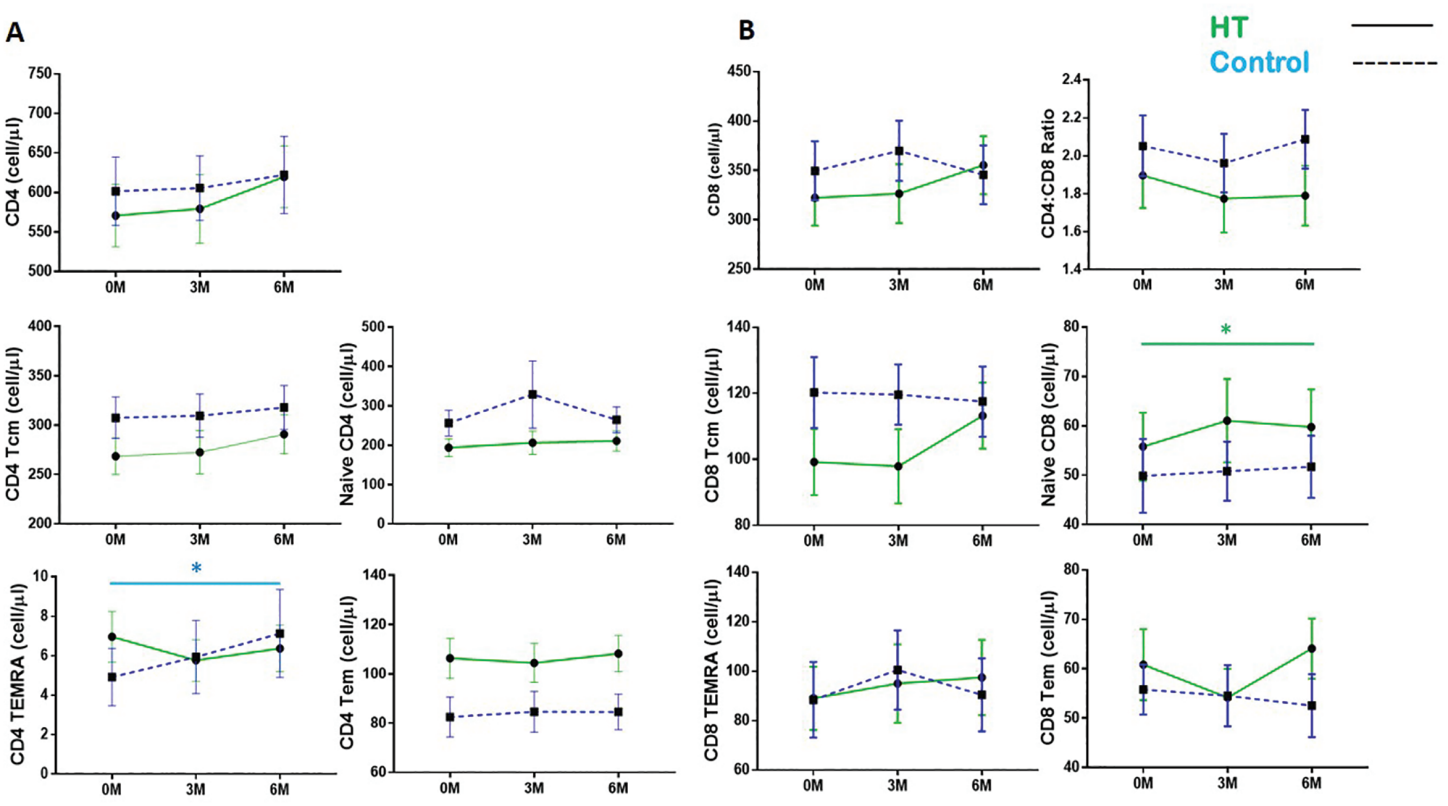
Naive CD8 T-cell numbers increase during horticultural therapy (HT). (**A**) Changes in CD4^+^ T-cell subsets, depicting naive, TCM, TEM, and TEMRA subsets. (**B**) Changes in CD8^+^ T-cell naive and memory subsets as well as CD4^+^/CD8^+^ T-cell ratios. Upper and lower horizontal bars indicate the standard error of the mean. Points are indicative of the mean cell count for each subset; waitlist (blue) and HT intervention (green). Friedman test was applied to HT or waitlist control groups to analyze differences within treatment groups over the 3 time points; *p-value < .05.

Guided by the initial t-SNE analysis, we proceeded to examine the kinetics of naive and TEMRA CD4^+^ and CD8^+^ T-cell subsets by manual flow cytometry gating (Supplementary Figure 1B and C). Unlike the t-SNE analysis, all participants who were present at all 3 allocated blood draws were included in the analysis (HT: *n* = 22; waitlist: *n* = 24). In combination with the use of Trucount beads, this strategy allows us a further scope of observing fluctuations in the absolute numbers of T-cell subsets within whole blood as compared to fluctuations in their proportions within T cells as in our t-SNE analysis. Here, we detected no significant differences in the kinetics of CD4^+^ and CD8^+^ T-cell numbers, as well as CD4:CD8 ratios between time points in both the HT and waitlist groups (Figure 3).

Next, CD4^+^ and CD8^+^ T cells were sorted into naive and Tcm, Tem, and TEMRA memory subpopulations using CD27 and CD45RA expression (Supplementary Figure 1B1 and C1): Naive (CD45RA^+^CD27^+^), TCM (CD45RA^−^CD27^+^), TEM (CD45RA^−^CD27^−^), and TEMRA (CD45RA^+^CD27^−^). Specific to CD4^+^ T cells, we observed that the numbers of CD4^+^ TEMRA significantly increased from baseline at both the 3M and 6M time points in the control group, but not in the HT group (Figure 3A). Concordant with our most prominent t-SNE observation, naive CD8^+^ T-cell numbers were also increased following HT but not in the waitlist control group (Figure 3B). The kinetics of CD8^+^ TEMRA were stable and comparable between the HT intervention and waitlist control groups over the 3 time points. The individual time data points that constitute the mean numbers shown in Figure 3 are presented in Supplementary Figure 3.

### HT Is Associated With a Reduction in the Proportion of CTLA4^+^ TEMRAs

Although there were no significant changes in the numbers of CD4^+^ and CD8^+^ TEMRAs within the HT group across the 3 time points, our initial t-SNE analysis reflected that dynamic changes exist within TEMRA subpopulations during the 6-month period of this study. Moreover, we observed an increase in CD4^+^ TEMRA cell numbers in the waitlist, but not the HT group. Separately, we observed an accelerated accumulation of CTLA4^+^ CD8^+^ TEMRAs in the waitlist but not the HT group at the 3M time point of this study. Since the study of total TEMRA numbers may not reflect dynamic changes within TEMRA subpopulations, we subdivided TEMRA CD4^+^ and CD8^+^ T cells into subsets based on their combinatorial expression of CD57, KLRG1, and CTLA4 (Supplementary Figure 1B and C).

**Figure 4.**
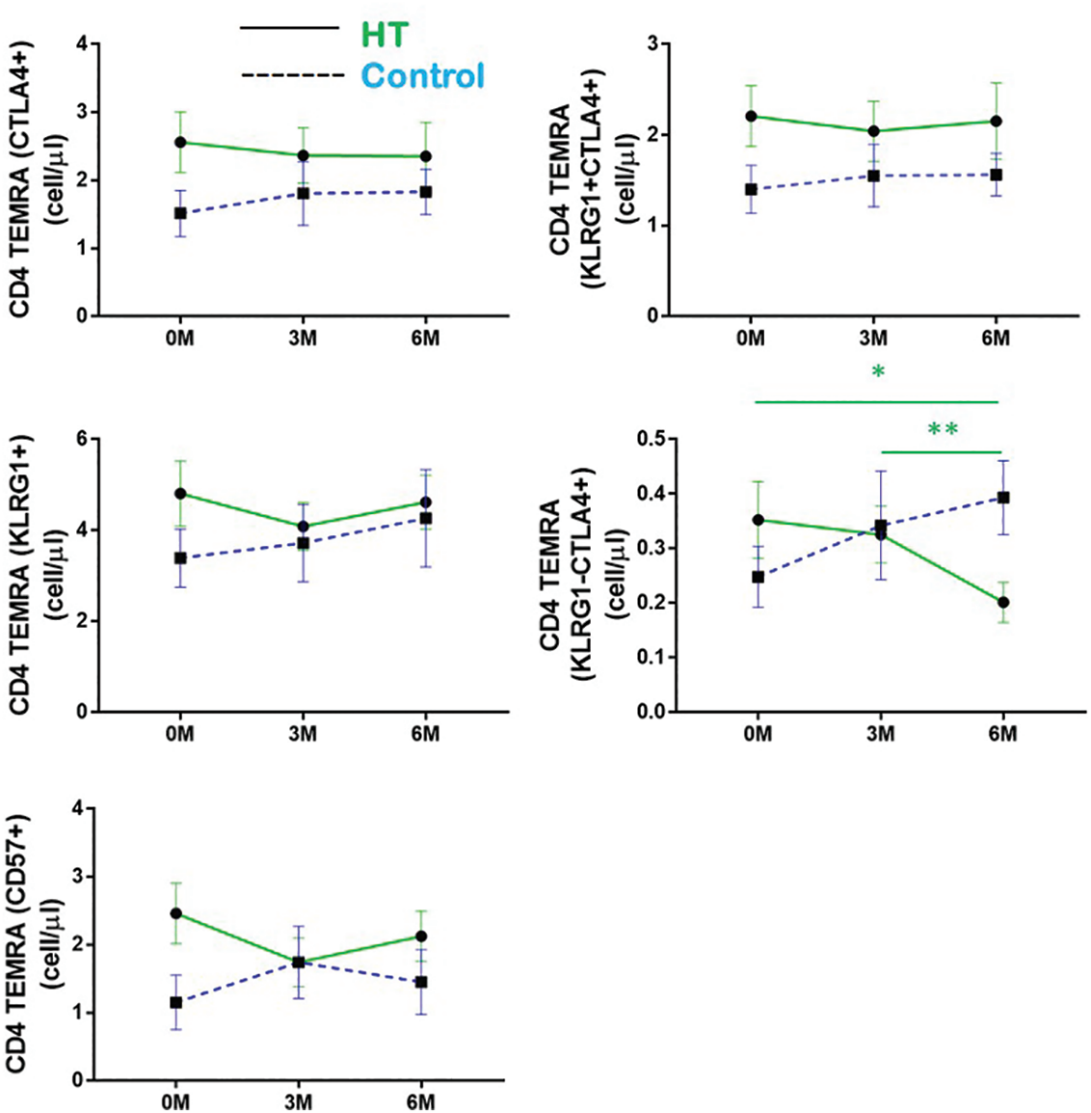
CD4 TEMRAs expressing CTLA4, KLRG1, and CD57 decrease in numbers during horticultural therapy (HT). Changes in the numbers of CD4 TEMRAs expressing CTLA4, KLRG1, and CD57 are individually shown, also shown are the numbers of KLRG1^−^CTLA4^+^ and KLRG1^+^CTLA4^+^ CD4 TEMRAs at each time point. Upper and lower horizontal bars indicate the standard error of the mean. Points are indicative of the mean cell count for each subset; waitlist (blue) and HT intervention (green). Friedman test was applied to HT or waitlist control groups to analyze differences within treatment groups over the 3 time points; *p-value < .05, ** p-value < .01.

While changes were not statistically significant, we observed a trend where the numbers of CD4^+^ TEMRAs individually expressing CTLA4, KLRG1, and CD57 were reduced from baseline at both the 3M and 6M time points within the HT group. However, we observed a striking decrease in the numbers of KLRG1^−^ CTLA4^+^ CD4^+^ TEMRAs when we considered the combinatorial expression of KLRG1 (Figure 4). On the contrary, within the control group, we observed an increase in the numbers of CD4^+^ TEMRA subsets expressing a combination of KLRG1, CD57, and CTLA4^+^ from baseline at the 3M and 6M time points. Although these changes were not statistically significant, their collective modulations likely contribute to the significant increase in total CD4^+^ TEMRA numbers within the waitlist group (Figure 3A). The individual time data points that constitute the mean numbers shown in Figure 4 are presented in Supplementary Figure 4.

### CTLA^+^ CD8^+^ TEMRAs Decrease in Proportion Following HT

**Figure 5.**
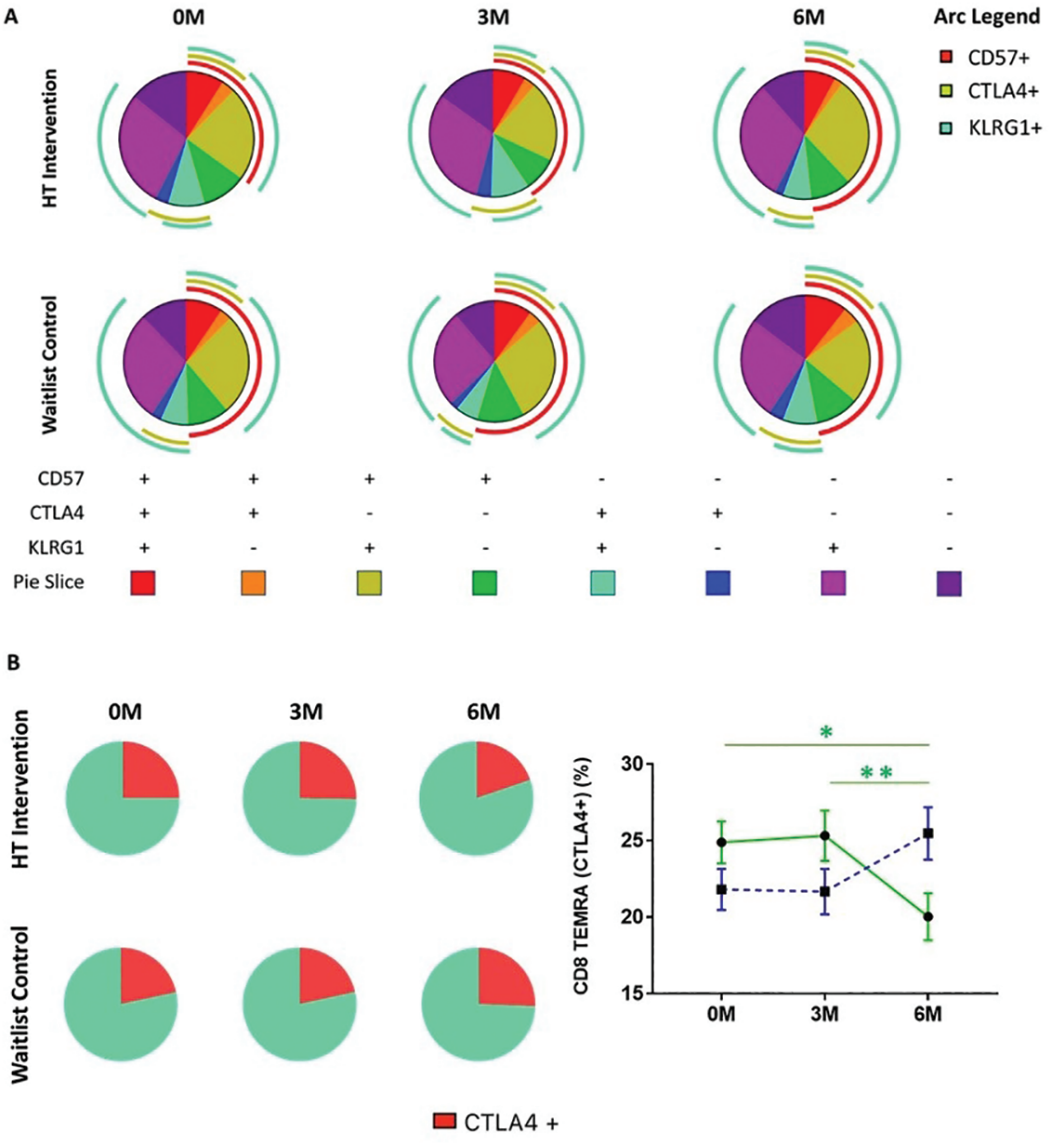
Reduction of CTLA4^+^ expressing CD8^+^ TEMRA cells observed in horticultural therapy (HT) intervention group. (**A**) Pie chart indicating the proportion of 8 different CD8 TEMRA subsets based on the combinatorial expression of CD57, CTLA4, and KLRG1. Arcs indicate specific marker expression on CD8^+^ TEMRA cells. (**B**) Pie chart depicting changes in the proportions of CTLA4^+^ and CTLA4^−^ CD8^+^ TEMRA cells at each time point (right). Changes in the percentages of CTLA4^+^ CD8 TEMRAs within total CD8 TEMRAs. Friedman test was applied to HT or waitlist control groups to analyze differences within treatment groups over the 3 time points; *p-value < .05; **p-value < .01.

Consistent with data described by others, we observed much larger numbers of CD8^+^ TEMRAs than CD4^+^ TEMRAs in our older adults cohort (Supplementary Figure 4) (13,38). While this was not statistically meaningful due to low numbers of CD4^+^ TEMRAs, we were able to study the kinetics of all 8 permutations of CD8^+^ TEMRAs expressing a combination of CD57, CTLA4, and KLRG1 using SPICE. The proportions of CD8^+^ TEMRA subsets at each time point are presented as pie charts in Figure 5A. Supplementary Table 3 shows the statistical comparisons of all pie charts using Monte Carlo simulation, which determines if each pie chart differed in their proportions of TEMRA subsets from another.

We observed opposite trends in the modulations of sectors corresponding to CD57^−^KLRG1^−^CTLA4^+^ (dark blue) and CD57^−^KLRG1^+^CTLA4^+^ (cyan) in the HT and waitlist group, in that both sectors were, respectively, reduced and increased in the HT intervention and waitlist groups. While the latter modulations in these specific sectors were not found to be statistically significant across time points within each study group, we observed a significant decrease in CTLA4^+^ CD8^+^ TEMRAs in the HT group when all CTLA4^+^ CD8^+^ TEMRA sectors were grouped (Figure 5B).

### Correlation of CD8^+^ T-Cell Populations With IL-6

**Figure 6.**
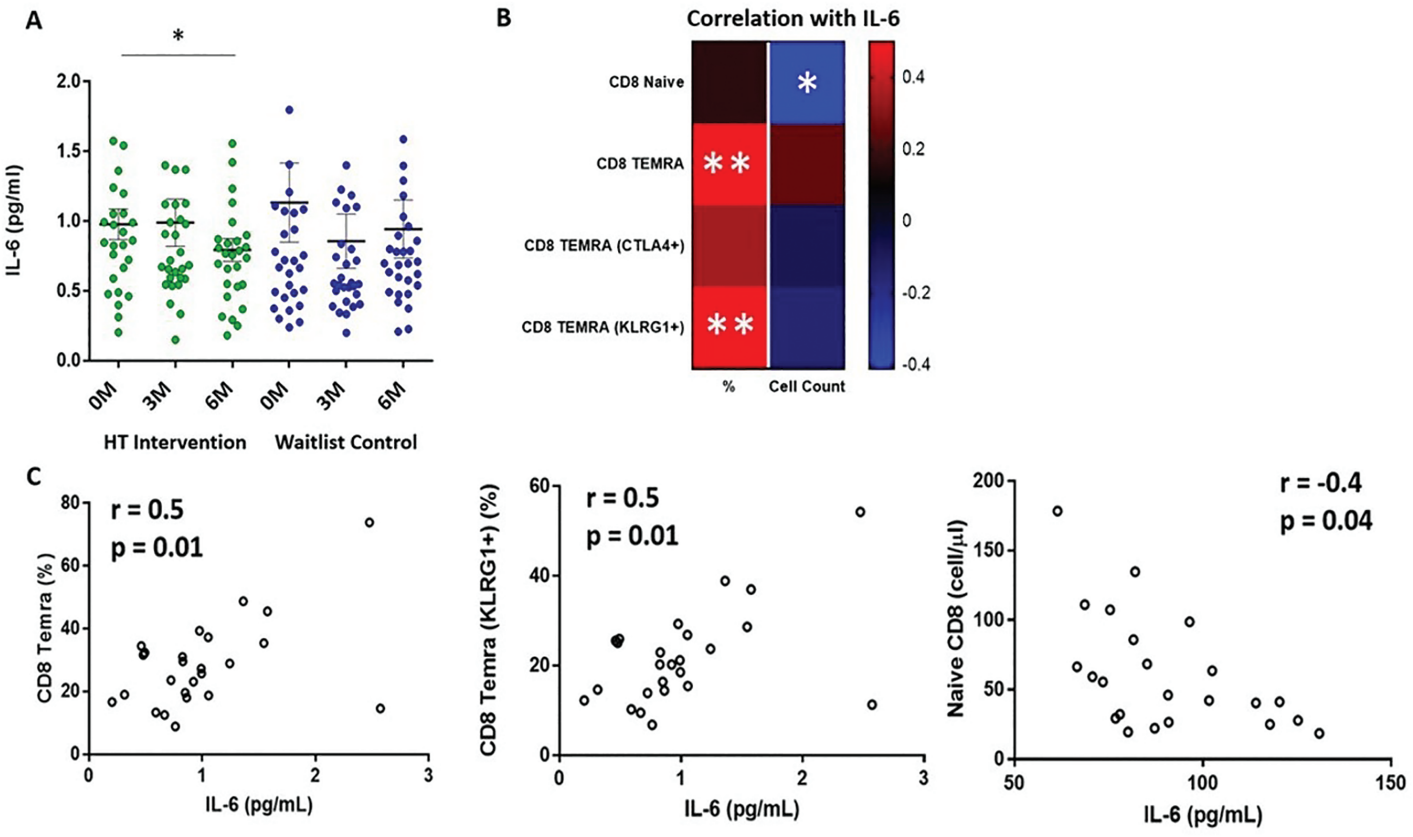
Correlations between plasma IL-6 levels and naive and TEMRA CD8 T-cell subsets. (**A**) Decrease in plasma IL-6 levels observed in horticultural therapy (HT) intervention group compared to waitlist control group (60). (**B**) Heatmap displaying the correlation between the percentages of CD8 T-cell subsets to plasma IL-6 levels of participants in the HT group. Percentages of (**C**) CD8^+^ TEMRA and CD8^+^ TEMRA (KLRG1^+^) are positively correlated to plasma IL-6 levels; while naive CD8^+^ T-cell counts are negatively correlated to plasma IL-6 levels; *p-value < .05; **p-value < .01.

As IL-6 was the only inflammatory marker that was found to be significantly modulated during HT (Figure 6A), we looked for correlations between IL-6 levels and the percentages of naive and TEMRA CD8^+^ T-cell subsets in the HT group at the 6-month time point to determine if the modulations in these subsets could be related to changes in IL-6 levels during HT. We observed significant positive correlations between IL-6 levels and the percentages of CD8^+^ TEMRA (Figures 6B and C) within the HT group at 6M. Furthermore, we also observed an inverse correlation between IL-6 levels and CD8^+^ naive T-cell numbers within the HT group at 6M. While IL-6 levels were positively associated with the percentages of CTLA4^+^ CD8^+^ TEMRAs, this observation was not statistically significant. The latter suggests that the decrease in CTLA4^+^ CD8^+^ TEMRAs during HT may be more closely related to the modulation of immunological factors other than IL-6. Altogether, the data suggest that the immunomodulatory benefits of HT, with respect to reduced CD8^+^ T-cell exhaustion, were associated with decreased IL-6 levels within the HT intervention group.

## Discussion

While the effects of HT on self-reported measures of mental health have been relatively well studied, evidence of the effects of HT on the immune system remain scarce. In this study, we demonstrate for the first time that following a 6-month HT intervention program, HT was associated with reduced T-cell exhaustion and inflammaging in community-dwelling older adults. We showed that HT was associated with increased numbers of naive CD8^+^ T cells and reduced numbers of CTLA4-expressing TEMRA T cells. Furthermore, the frequencies of these subsets were associated with IL-6 levels, which was reduced during HT, at the 6M time point. Altogether, our findings suggest that reduced IL-6 levels during HT may contribute to enhanced naive CD8^+^ T-cell hematopoiesis and a decrease in the proportions of exhausted CTLA4-expressing CD8^+^ T cells following HT (Supplementary Figure 5). By demonstrating its immunological impact, we complement previous studies that have demonstrated health benefits of HT, such as improved well-being and cognitive function (39,40).

Markers of immunosenescence are often associated with functional decline and age-associated morbidities and mortality (41). In particular, T-cell exhaustion and senescence are major obstacles to the resolution of infections and cancer (6). The expression of CTLA4 in T cells is significantly correlated with aging and contributes to age-related T-cell anergy (42). Furthermore, PD-1 and CTLA4 repression in T cells represent major obstacles to the efficacy of CAR-T treatment, and PD-1 and CTLA4 blockade by monoclonal antibodies have been intensively explored in cancer treatment (43). Pertinently, HT has already been institutionalized in some hospitals to reinforce mental fortitude in cancer patients and our findings suggest that by lowering the expression of exhaustion markers, HT may also be beneficial in promoting T-cell anti-tumor surveillance in older adults (44). More generally, since HT was beneficial in alleviating symptoms of T-cell senescence and exhaustion within the intervention arm, HT could serve as an attractive option to boost immunological health in the older adults.

Research has shown that serum IL-6 concentration increases with age and IL-6 is an established marker of inflammaging that is associated with morbidities and mortality in older adults (45). Produced by macrophages and monocytes at inflammatory sites, in the aged population, elevated IL-6 levels are associated with a higher risk of dementia, depression, rheumatoid arthritis, and cardiovascular disorders (46–48). While IL-6 is required during infection and injury to stimulate and activate host defenses through its pleiotropic effects on inflammation and immunomodulation, the aggressive secretion of IL-6 is known to contribute to chronic inflammation and associated pathologies (49). Inflammaging has been proposed to be macrophage-centered and could result from a lower threshold for innate cell activation or the increased presence of microbial products within the older adults’ circulation (50,51). Aside from changes in the frequencies of T-cell subsets, we observed that older adults from the HT group also had lower serum IL-6 levels as compared to the waitlist control group. The latter suggests that HT may be beneficial in delaying inflammaging. Importantly, we observed that post-HT IL-6 levels were associated with reduced frequencies of naive CD8^+^ T cells and higher frequencies of TEMRA CD8^+^ T cells in HT participants. Since IL-6 promotes progressive T-cell differentiation, including the proliferation of CD8^+^ T cells—this mechanism could explain the observed associations and support our hypothesis that some of the immunological benefits of HT observed in our study may be attributed to its relationship with reduced IL-6 levels (52). Notwithstanding these associations, the reduction of serum IL-6 levels in the HT intervention group alone suggests that HT may be beneficial in the prevention and treatment of age-related morbidities.

We recognize the limitations of this study that are inherent in the small sample size and high frequency of female participants. The sample size used in this study was modest, and we encourage larger replications of this study to confirm the immunological benefits of HT. We also acknowledge that the benefits of HT in our study may be specific to our Asian cohort of community-dwelling older adults that consists of a majority of women, thus limiting generalizability to populations of a different demographic, particularly in terms of gender distribution, ethnic background, or infection history. Since hormonal differences have a bearing on immune constitution, future studies must include more balanced gender distributions to confirm that the immunological benefits of HT observed in this study are not biased towards females (53,54). Additionally, we acknowledge that the findings in our report may be sporadically observed due to extensive testing on multiple T-cell subsets, and will benefit from confirmation through reproduction in subsequent studies that focus on specifically analyzing the impact of HT on naive and CTLA4^+^ expressing TEMRA T-cell populations. Owing to the pilot and exploratory nature of this study, future studies can involve more participants to achieve greater statistical power.

Next, the design of our study does not allow us to identify specific behavioral factors within our HT intervention that contribute most strongly to the observed immunological benefits. These factors can include physical activity or socialization levels, which were not adequately controlled for in our waitlist control study design. Since the impact of HT on immune parameters, and the influence of physical activity on immunosenescence is a developing concept, this study was built on traditional HT protocols that have neglected to control for the positive influence of physical activity that was encouraged through walking activities within the HT intervention between waitlist and control groups (31,37–39,55). As a study that examines the impact of HT on immunological phenomena that can be affected by behavior (physical activity levels, diet etc.), future studies should control for these factors by either encouraging similar physical engagement within the waitlist group or collecting data pertaining to physical activity and dietary behavior—the latter so that their interaction with the effects of HT can be controlled during data analysis. We were also unable to determine whether improved social connectedness due to participation in group activities is a contributing factor to the observed immunological benefits of HT. Nonetheless, our previous analyses of this cohort support the notion that improved social connectedness does not account for the majority of the change in IL-6 within the HT group (56). Lastly, we note the lack of blinding of participants to the assigned interventions, functional data, or clinical outcomes reported in this study. Future trials should incorporate a longer follow-up duration and further include clinical and functional measures.

Despite these limitations, this study has addressed several critical shortcomings present in previous HT studies, and we have shown pilot data on the multiple the immunological impacts of HT. An important innovation of this HT study is its RCT and longitudinal design, which demonstrates stronger evidence of causality as compared to observational data (57). Nature-based interventions typically suffer from the lack of large, well-designed and longer-term trials (39). Through the addition of a waitlist control group, we have overcome a major limitation of previous HT studies that utilized a single-group design. We also examined the longer-term effects of HT by incorporating 3- and 6-month follow-ups, which enabled us to detect immunological changes that require more time to manifest. Furthermore, we also examined a large profile of biological markers utilizing both flow cytometry and ELISAs, including an extensive amount of circulating and aging-related T-cell subpopulations with distinct cellular surface biomarkers. This comprehensive profiling of both immunosenescence and inflammaging in older adults enabled us to pinpoint the specific effect of HT on particular facets of immune functions, laying the groundwork for future investigations on the immunological effects of HT.

The geroscience hypothesis proposes that biological—rather than chronological—aging is a common and major risk factor for developing chronic diseases and geriatric syndromes, and the use of senolytics has been proposed to delay these developments (58). However, due to socioeconomic reasons, these interventions are not popular at the population level. Furthermore, research into the potential efficacy of psychosocial interventions, such as HT, in delaying biological aging has seen little interest (59). We are optimistic that data presented here may serve as a platform that encourages larger-scale investigations that seek to confirm the immunological and anti-aging benefits of HT, as nature-based activities can be a practical strategy to improve immunological fitness in the elderly. In summary, through a 6-month HT intervention program, we demonstrated for the first time, that HT is beneficial in reducing the levels of biomarkers associated with T-cell senescence, exhaustion, and inflammaging. Further studies are needed to discern whether the immunological benefits observed in this study are a function of the horticultural experience per se or due to increased levels of socialization or physical activity during the intervention period. Nonetheless, HT can be an attractive way to increase physical activity levels within the older adults, which carries its own immunological benefits (61). With the growing abundance of green spaces available worldwide—HT can be a safe, feasible, and cost-effective strategy to promote healthy aging within rapidly aging societies.

## Supplementary Material

glaa271_suppl_Supplementary_Figures_Table_FINAL

## Data Availability

The data that support the findings of this study are openly available in Mendeley Data at http://dx.doi.org/10.17632/rwk76gcxt8.1, v1.
