# Supplementary material for: Horticultural Therapy Reduces Biomarkers of Immunosenescence and Inflammaging in Community-Dwelling Older Adults: A Feasibility Pilot Randomized Controlled Trial
Source: J Gerontol A Biol Sci Med Sci. 2020 Oct 18;76(2):307–17. doi: 10.1093/gerona/glaa271 (PMC7812436; doi:10.1093/gerona/glaa271)
Supplement: glaa271_suppl_Supplementary_Figures_Table_FINAL [file glaa271_suppl_supplementary_figures_table_final.docx]

**Supplementary Figures & Table**

**Supplementary Table 1. Planned activities for the 6 months of HT intervention**

| **Session** | **Topic** | **Activity** | **Venue** |
| --- | --- | --- | --- |
| 1 | Introduction | 1. Familiarisation with research centre 2. Group formation 7/group 3. Indoor Gardening basics | Research Centre |
| 2 | Introduction | 1. Garden familiarisation 2. Vegetables growing brief 3. Sowing vegetables seedlings | Chinese Garden |
| 3 | Wetland Walk | 1. Park amenities familiarization 2. Interpretive walk 3. Reflection | Sungei Buloh Reserve |
| 4 | Introduction | 1. Briefing for vegetables maintenance 2. Weeding and fertilizing vegetables plot | Chinese Garden |
| 5 | Nurturing | 1. Briefing on pressed flowers 2. Material preparation 3. Make pressed flowers card | Research Centre |
| 6 | Nurturing | 1. Garden maintenance briefing 2. Weeding, pruning, mulching garden/vegetables | Chinese Garden |
| 7 | Colour Walk | 1. Park amenities familiarization 2. Interpretive walk 3. Reflection | Singapore Botanical Gardens |
| 8 | Nurturing | 1. Vegetables maintenance 2. Compost making brief 3. Make compost | Chinese Garden |
| 9 | Harvest and Cook | 1. Harvest vegetables 2. Hands on preparation for food 3. Sharing of cooked vegetables | Research Centre |
| 10 | Harvest and Cook | 1. Seed sowing 2. Herbal plant brief 3. Herbal plants propagation | Chinese Garden |
| 11 | Festive Walk | 1. Park amenities familiarization 2. Interpretive walk 3. Reflection | Gardens by the Bay (Flower Dome) |
| 12 | Harvest and Cook | 1. Community Garden tour 2. Plant care tips for herbs and plants that will be brought home 3. Reflection | Chinese Garden |
| 13 | Healing Walk | 1. Park amenities familiarization 2. Interpretive walk 3. Reflection | Botanical Garden – Healing Garden |
| 14 | Gardening | 1. Briefing 2. Create a culinary garden 3. Maintenance tips | Chinese garden – culinary garden creation |
| 15 | Nature Walk | 1. Park amenities familiarization 2. Interpretive walk 3. Reflection | Gardens by the Bay (Cloud Forest) |

Note: Intervention sessions 1 to 12 were conducted on a weekly basis while sessions 13 to 15 were conducted once a month

**Supplementary Table 2: Antibody master mix used for immunophenotyping**


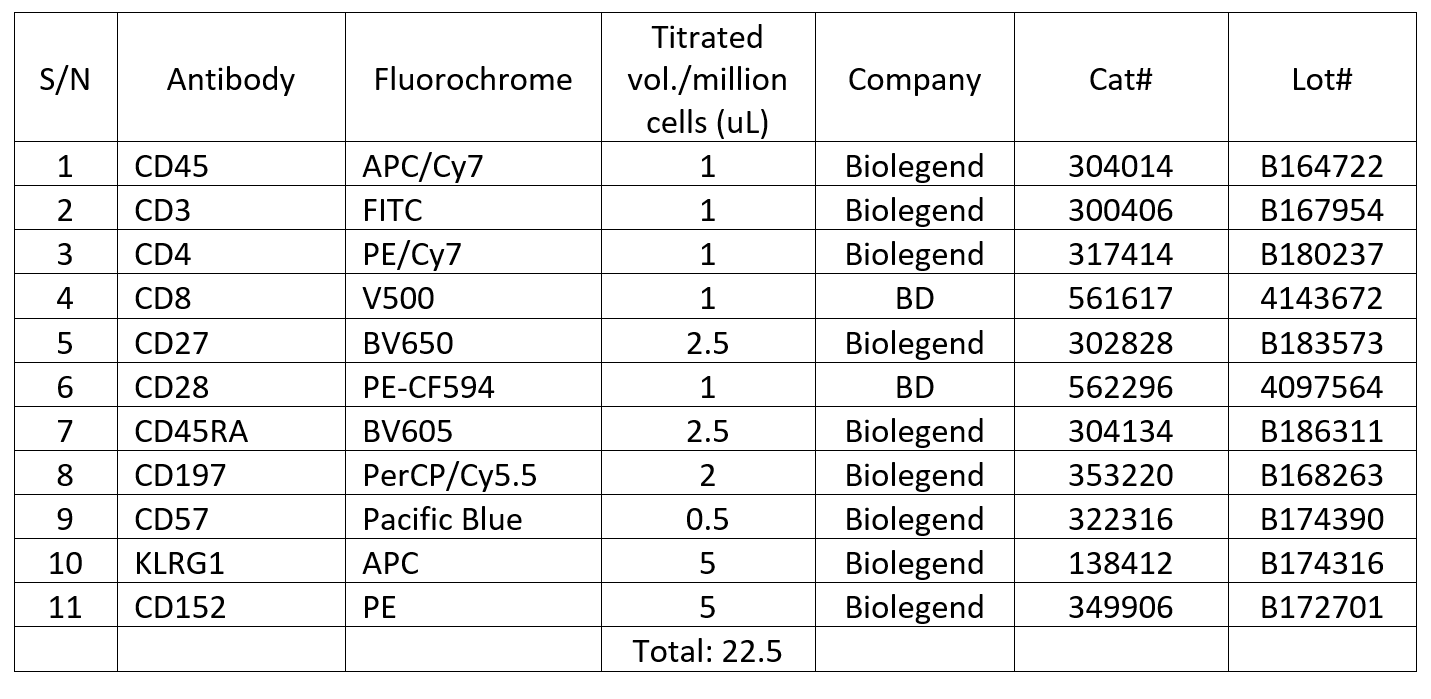


**Supplementary Table 3.** Statistical significance of SPICE analysis between the various time-points within the HT Intervention Group and Waitlist Control Group and across the two groups

**
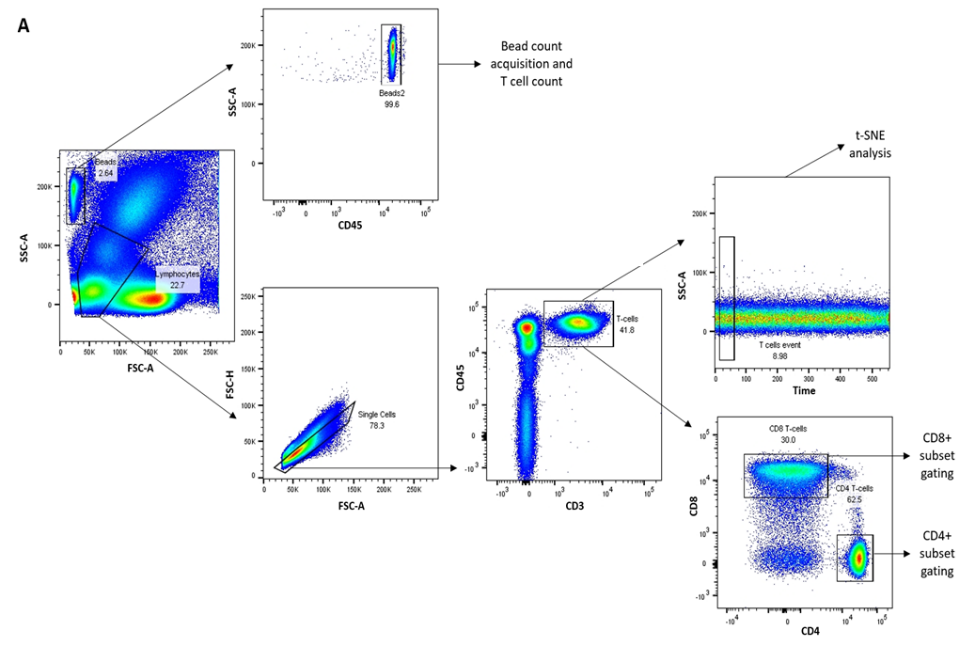
**


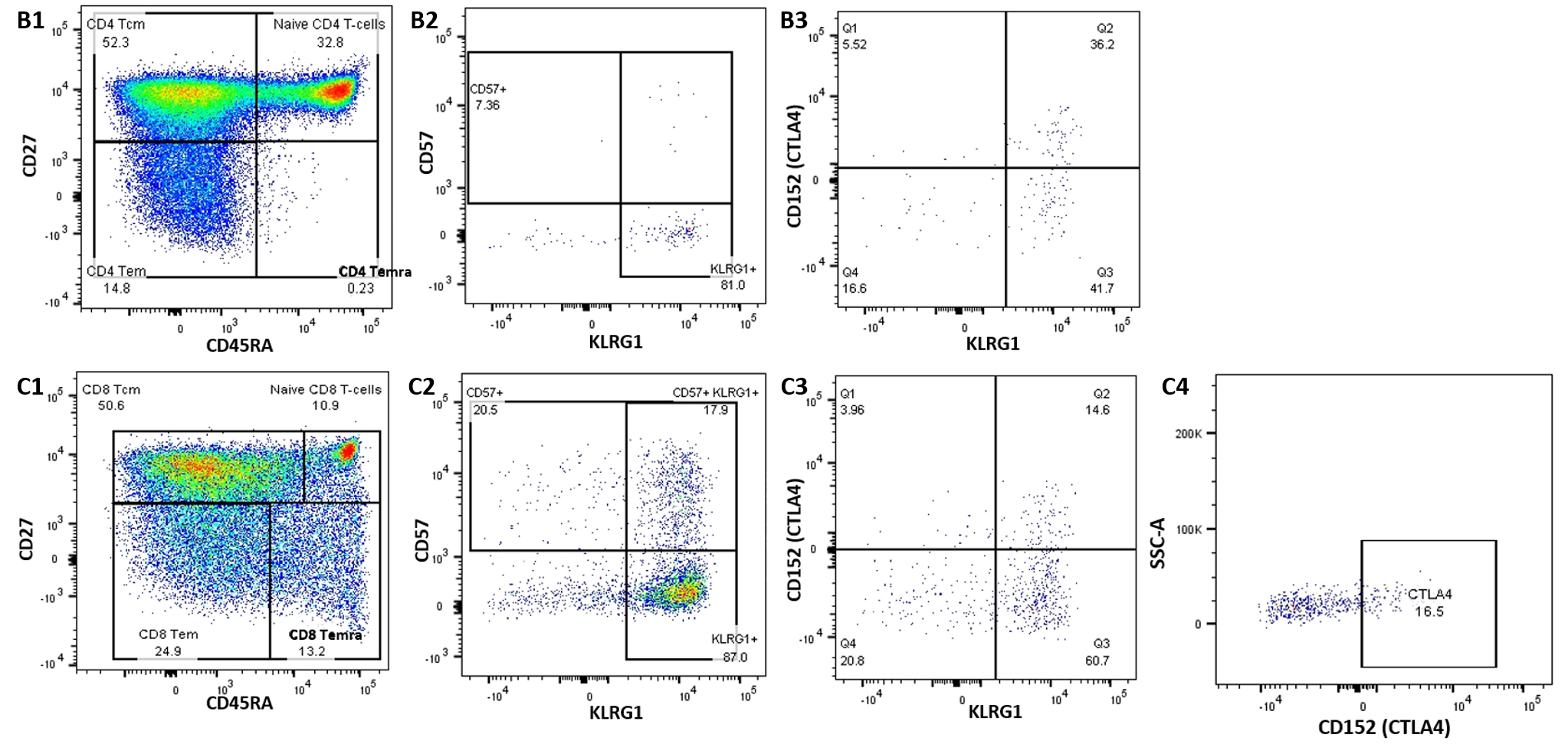


**Supplementary Figure 1. Flow cytometry gating strategy.** (A) Preliminary gating for bead count and T cell subsets. 10,000 T cell events from 10 random participants from both HT intervention and waitlist control groups were gated as shown at each time point and concatenated for t-SNE analysis. Thereafter, the gating strategy for CD4 and CD8 T-cells in the immunophenotyping of participants who attended all blood collection timepoints (HT: n=22, Watilist: n=24) are shown in B and C respectively. B1 and C1: gating of naïve, central (Tcm) and effector (Tem) memory as well as TEMRA subsets. TEMRA CD4 and CD8 T-cells were subsequently gated as depicted (CD4 T-cells: B2 – B3; CD8 T-cells: C2 – C4) to study the proportion of TEMRA cells expressing CD57, KLRG1 and CTLA4.

**A**


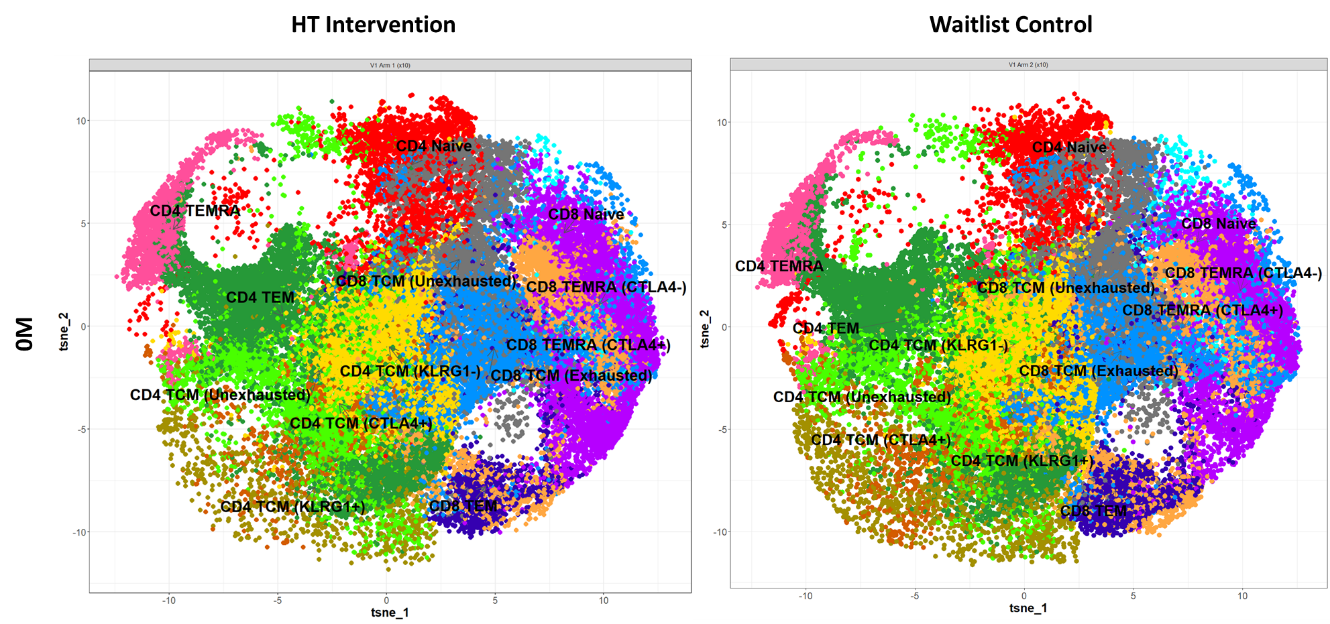


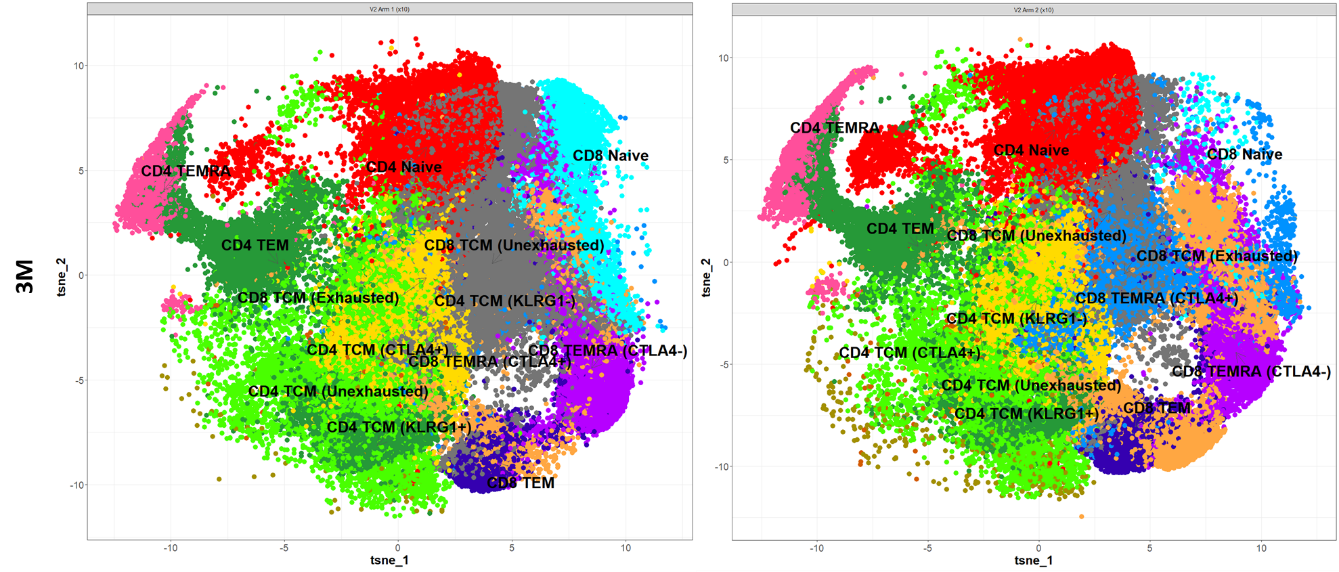


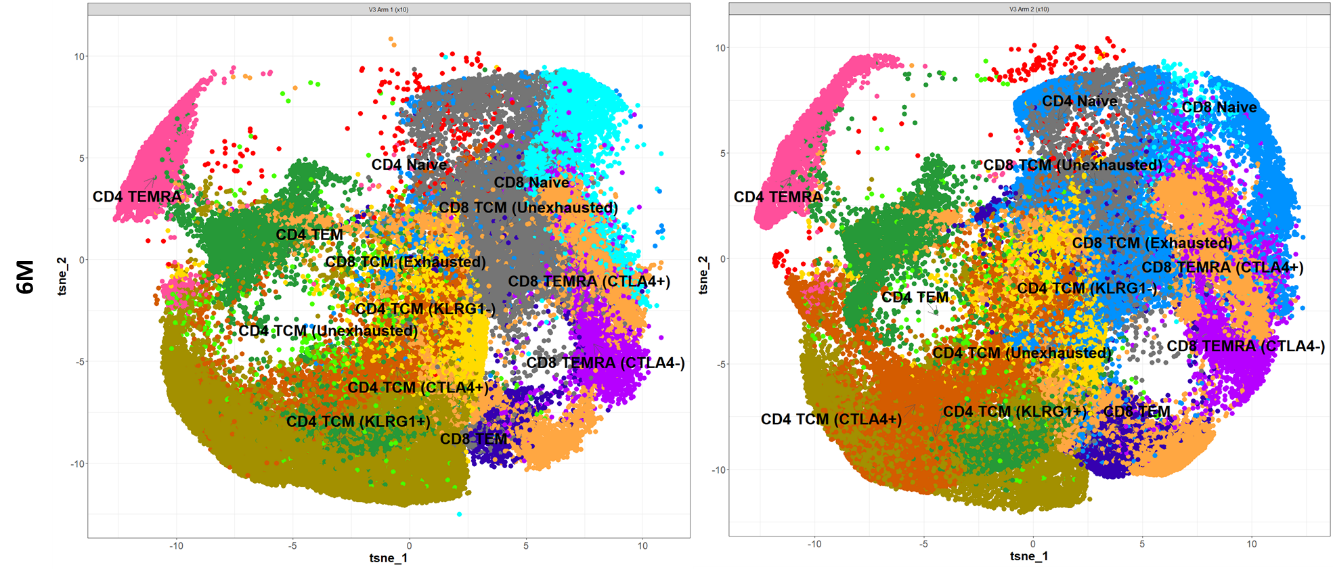


**B**


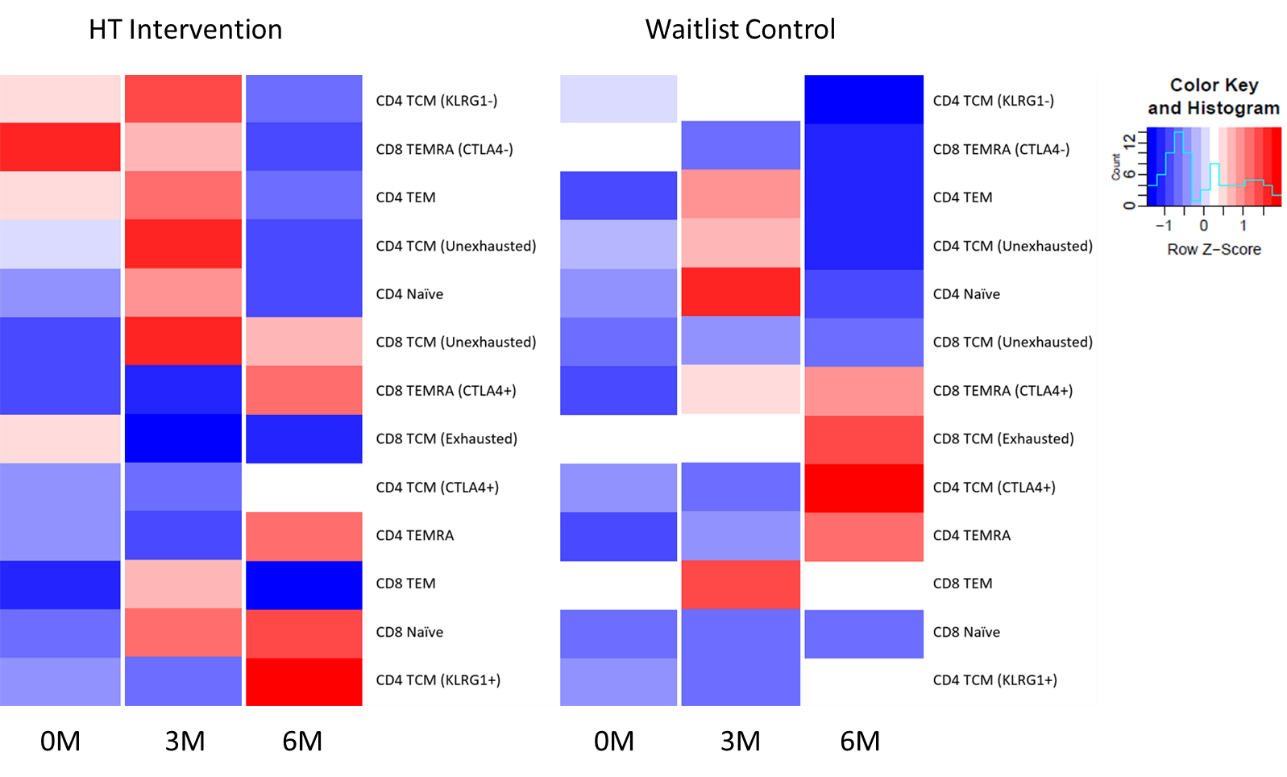


**Supplementary Figure 2. Kinetics of T-cell clusters at each time point.** (A) Time-point specific cluster plots for both allocation arms were generated by t-SNE, including 10,000 T-cells from ten randomly selected donors that were pooled together for each allocation arm (total of 100,000 T-cells per plot). 13 phenotypically distinct T cell clusters were obtained. (B) Kinetics of 13 T-cell clusters expressed as a heatmap (row normalisation).


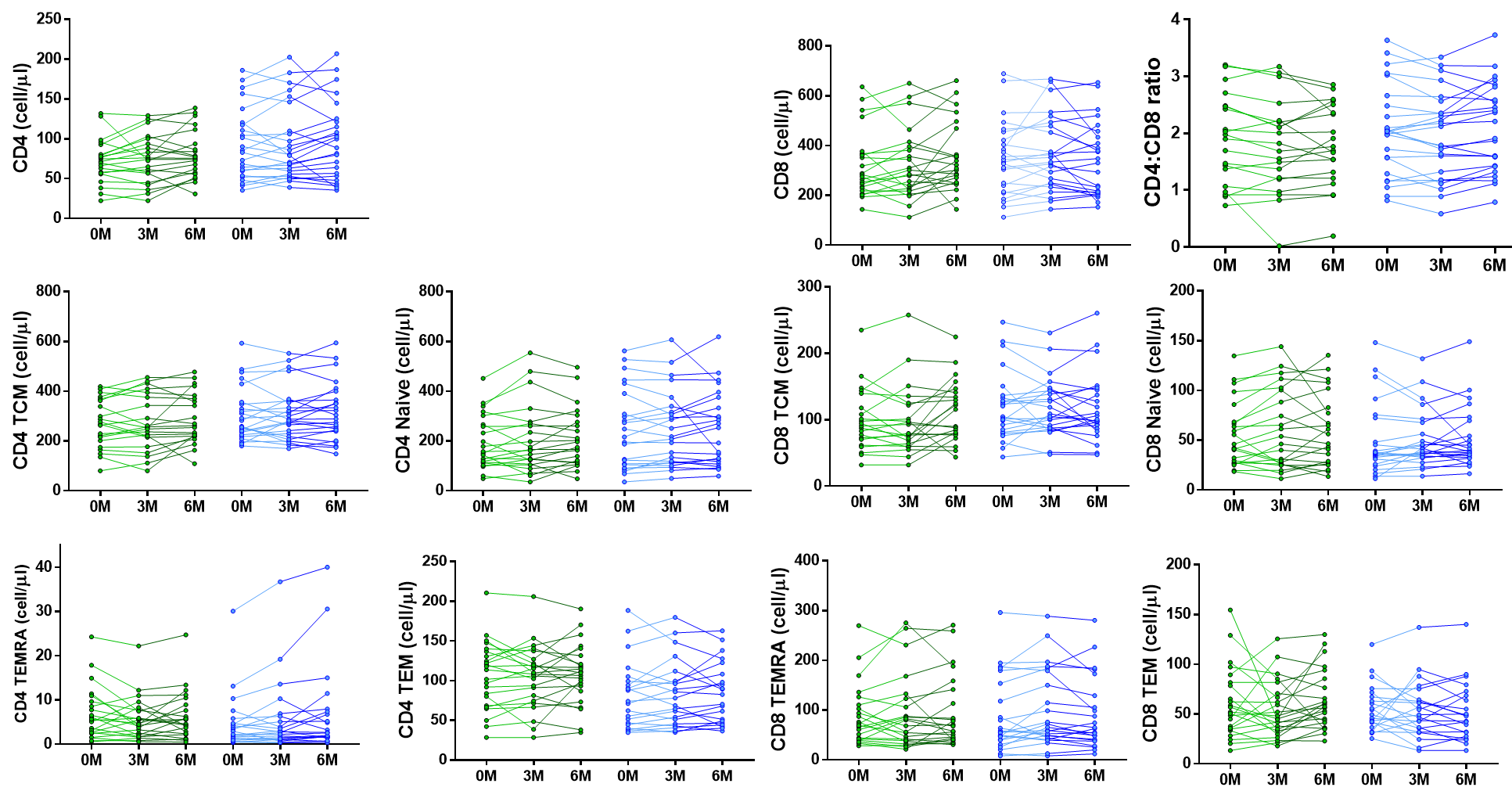


**B**

**A**

**Supplementary Figure 3: Naive CD8 T-cell numbers increase during HT.** (A) Changes in CD4+ T cell subsets, depicting naïve, Tcm, Tem and TEMRA subsets. (B) Changes in CD8+ T cell subsets and CD4+/CD8+ T cell ratios. Upper and lower horizontal bars indicate the standard error of the mean; waitlist (blue) and HT intervention (green).


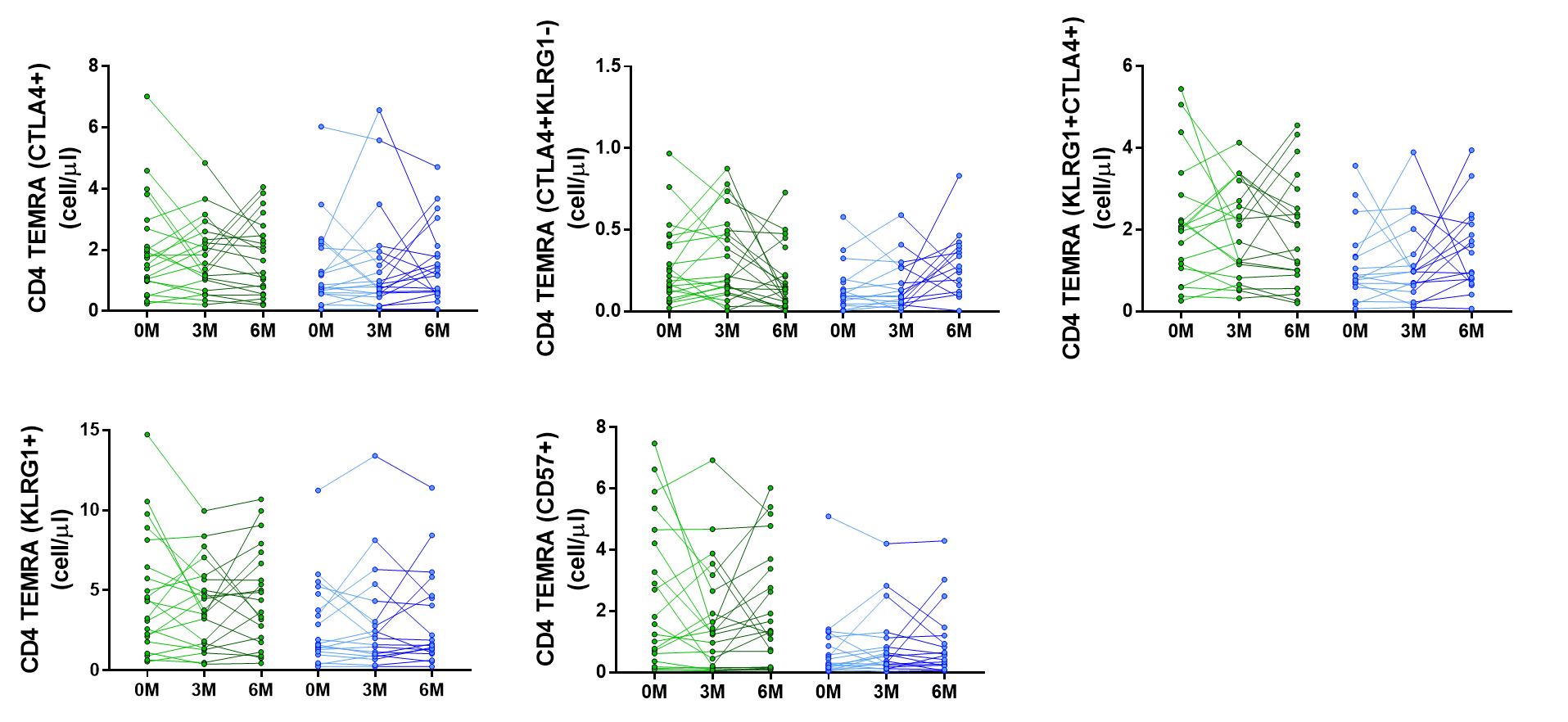


**Supplementary Figure 4: CD4 TEMRAs expressing CTLA4, KLRG1 and CD57 decrease in numbers during HT.** Changes in the numbers of CD4 TEMRAs expressing CTLA4, KLRG1 and CD57 are shown for each participant, together with the numbers of KLRG1-CTLA4+ and KLRG1+CTLA4+ CD4 TEMRAs at each time point; waitlist (blue) and HT intervention (green).

**
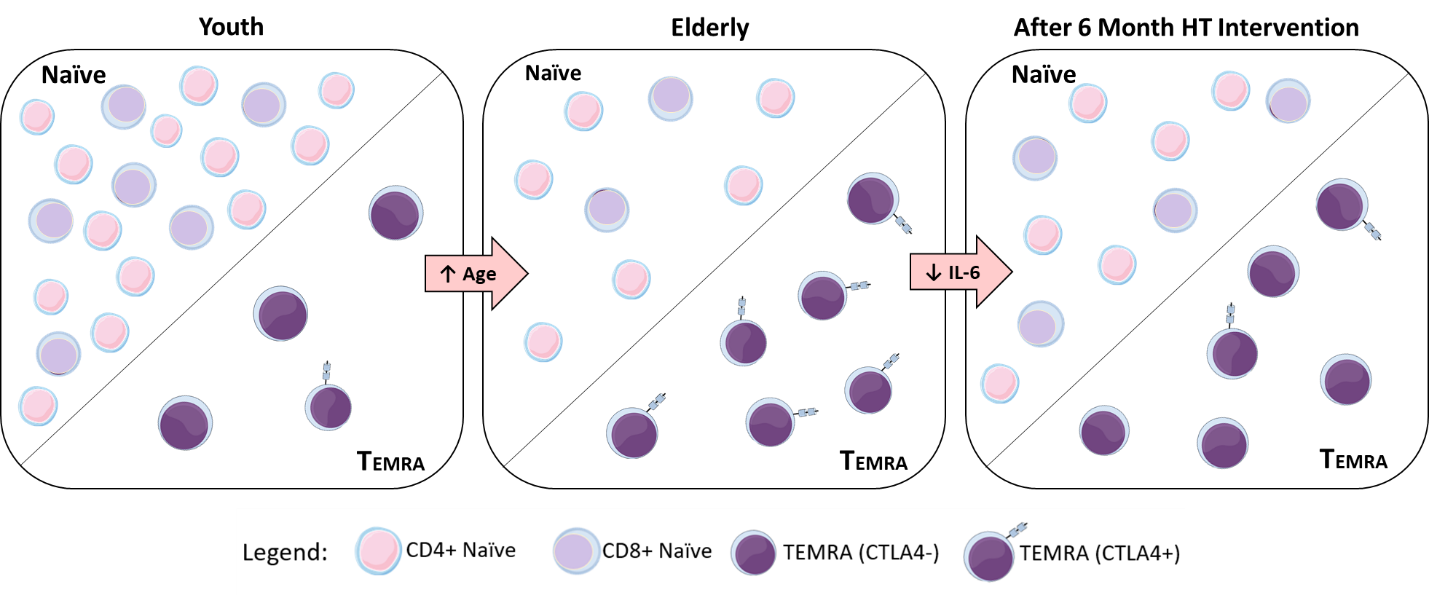
**

**Supplementary Figure 5: Changes in naïve and TEMRA T cell profiles with age and a proposed model for the effects of HT on both TEMRA cells and IL-6 levels.** (Left to right) Aging results in the loss of naïve CD8 and CD4 T cells as well as the accumulation of CTLA4+ expressing TEMRAs. An amelioration of these changes was observed after a 6M HT intervention in association with decreased IL-6 levels.
